# Caring for persons with drug use disorders in the Yangon Region, Myanmar: Socioeconomic and psychological burden, coping strategies and barriers to coping

**DOI:** 10.1371/journal.pone.0258183

**Published:** 2021-10-07

**Authors:** Khin Zar Khaing Thein, Chantal Herberholz, Win Pa Sandar, Yadanar

**Affiliations:** 1 National Health Laboratory, Dagon Township, Yangon, Myanmar; 2 Centre of Excellence for Health Economics, Faculty of Economics, Chulalongkorn University, Bangkok, Thailand; 3 Department of Health Behaviour and Communication, University of Public Health, Latha Township, Yangon, Myanmar; 4 Department of Health Policy and Management, University of Public Health, Latha Township, Yangon, Myanmar; South African Medical Research Council, SOUTH AFRICA

## Abstract

Drug use disorder (DUD) is a serious health condition that imposes a heavy burden on the persons who have a drug addiction experience and their families, especially in countries, such as Myanmar, where few formal support mechanisms are in place and repressive drug laws exacerbate the situation. Yet, in Myanmar, little is known about how informal caregivers are affected. This qualitative study aims at exploring the socioeconomic and psychological burden that informal caregivers in Myanmar encounter, coping strategies they employ, as well as barriers to coping they face. Thirty primary informal caregivers were chosen purposively from a mental health hospital in Yangon for in-depth interviews. The recorded interviews were transcribed and the data were analysed using framework analysis. The results revealed that financial constraint, income loss, social limitation and negative impact on family cohesion are important dimensions of socioeconomic burden, whereas sadness, anger, helplessness, worry, fear and guilt are the main psychological distress factors encountered by caregivers of persons with DUD. Key coping strategies employed by caregivers include religious coping, financial coping, acceptance and planning. Moreover, perceived stigma towards persons with DUD and their caregivers was very high and caregivers received hardly any social support, inter alia because of the country’s drug law which reinforces stigma and discrimination. Neither the government nor any other organization in Myanmar provided financial support to the caregivers. The results of this study showed that caring for persons with DUD has devastating effects on caregivers and their families. While the 2018 National Drug Control Policy can potentially help alleviate the burden on substance users and their families, further amendments of the existing drug law are urgently needed. Moreover, strengthening prevention and harm reduction approaches, improving treatment and rehabilitation services, as well as stigma-reducing educational campaigns should be considered a priority.

## Introduction

Drug use disorders (DUD) have become a major public health concern, increasing morbidity and premature mortality, and the burden of DUD has tremendous negative consequences for persons using drugs, their caregivers and families, as well as the society [1]. DUD are mental and behavioural disorders caused by the use of psychoactive and dependence-producing drugs [2, 3]. Almost 13 percent of adults worldwide who used drugs in the previous year, that is about 0.7 percent of the global adult population (aged 15 to 64), suffered from DUD in 2017 [4]. The global burden of disease attributable to drug use and drug use disorders was estimated to be 31.8 million and 20.4 million disability-adjusted life years in 2016, respectively [5]. Not captured by these health loss estimates are related economic and social consequences, which are substantial. In the United States, the impact of opioid use disorder and fatal opioid overdose in terms of healthcare, substance abuse treatment, criminal justice, lost productivity, reduced quality of life and premature mortality costs, for example, was estimated to be about 1.02 trillion US dollars in 2017 [6].

For most individuals with DUD, the family is the predominant source of support and the primary caregiving role is often assumed by a family member [7–9]. The impact felt by caregivers as a result of caregiving, the caregiver burden, is a multidimensional concept comprising social, economic, physical and emotional issues [10, 11]. In the literature, a distinction is made between objective and subjective burden [12, 13]. While the former captures how caregiving disrupts a caregiver’s life (that is, the socioeconomic burden comprising for example economic loss, social limitation, family conflict), the latter refers to the psychological burden. From a psychological perspective, the stress process associated with caregiving can be separated into four domains, namely the background and context of stress, primary and secondary stressors, mediators of stress such as coping and social support, as well as outcomes of stress [14]. Primary stressors are stressors that arise from the need of the patient, overload and relational deprivation, while secondary stressors comprise role strains (for example, economic problems, social limitations, family conflict) and intrapsychic strains (for example, loss of self-esteem and mastery) [14]. Ethnicity and cultural values also influence the stress process, especially through their effect on mediator variables [15–17]. Caregivers’ appraisal of caregiving stressors may result in negative or positive experiences of caregiving and affect their physical and psychological health [18, 19]. Studies that focus on the burden of caring for family members with mental illnesses such as bipolar and affective disorders, depression and schizophrenia, as well as the determinants thereof, have consistently shown that caregiving generates a burden, with severe negative effects on caregivers’ well-being across various dimensions [20–30]. The presence of caregiver burden was also confirmed in studies that specifically focus on DUD or co-occurring substance and mental disorders [7, 31]. In fact, the caregiver burden from caring for adolescents in treatment for substance use disorders and adolescents with mental health problems was found to be similar [32].

To mediate the effects of caregiving, active coping (i.e. seeking to confront and reduce stressors) such as performing religious activities, accepting the patient’s condition, planning for the future, responding positively to the current situation and avoidant coping (i.e. seeking to circumvent stressors) such as giving up caring are commonly used by caregivers of persons with DUD and severe mental disorders [28–31, 33, 34]. Stigma, lack of support, and lack of coping resources more generally, are identified in the literature as major barriers to coping [31, 35, 36].

The literature on substance users and DUD in South and Southeast Asia is scarce [35, 37, 38] and the evidence for Myanmar is limited to one policy briefing paper, which discusses the effects of the country’s harsh drug law enforcement practices on substance using persons and their families and argues that these increase stigma [39]. Myanmar is the second largest producer of opium and one of the largest producers of methamphetamines worldwide [4, 40, 41]. Due to the criminalisation of drug use, official statistics on drug users in Myanmar are not available, but drug problems are believed to be widespread [39]. In 2018, the 1993 Narcotic Drugs and Psychotropic Substances Law was amended and the country’s first National Drug Control Policy was released [42]. While the National Drug Control Policy, which was developed with support from the United Nations Office on Drugs and Crime (UNODC), focuses on public health and suggests decriminalizing drug use, the amended 1993 Narcotic Drugs and Psychotropic Substances Law, however, continues to impose harsh prison penalties on substance users [43].

Given that the National Drug Control Policy was only announced in 2018 and is not yet supported by an adequate legal framework, DUD in Myanmar is still a very sensitive issue. Hence, there are no studies that focus on DUD and the burden endured by caregivers of persons with DUD. This study, therefore, aimed at analysing the socioeconomic and psychological burden of caregivers. It also explored the coping mechanism used by caregivers to tackle the problems they encountered when caring for people with DUD, as well as barriers to coping.

## Materials and methods

This study employs a qualitative research design, using data collected through in-depth interviews.

### Participant recruitment

Participants for this study were recruited at the Drug Dependency Treatment and Research Unit (DDTRU) and Unit III of Yangon Mental Health Hospital (YMHH), one of the two tertiary-level mental health hospitals in Myanmar. Overall, there are 26 major and 47 minor drug treatment centres (DTC) in Myanmar, as well as 51 methadone maintenance therapy sites according to the Central Committee for Drug Abuse Control (CCDAC) [41]. Major DTC are attached to mental health or general hospitals and provide inpatient services, while minor DTC offer outpatient services [44]. Most persons who experience heroin dependence are at the DDTRU, whereas most persons who use amphetamines are treated at Unit III. The YMHH is located in the East Dagon Township, about thirty kilometres away from Yangon’s city centre. Thirty primary informal caregivers of persons with DUD who received treatment at the YMHH between April 2019 and July 2019 were chosen purposively, based on a priori determined eligibility criteria. Drawing on Goossens, Van Wijngaarden, Knoppert-Van Der Klein, & Van Achterberg [45], informal caregiver was defined as the person who has a strong relationship with the affected family member and provides most care, but is not a professional caregiver. Eligibility criteria included (i) being a primary family (informal) caregiver of a family member with DUD, (ii) co-residing with the family member, (iii) being 18 years and above, and (iv) having at least one year of caring experience. Caregivers of drug using family members who were participating in a rehabilitation and social reintegration programme at one of the country’s six rehabilitation centres [46], which also offer support services for caregivers, and paid caregivers were excluded. Information about patients’ background and medical history, as well as information about informal caregivers who accompanied their family member was obtained from registration records, psychiatrists and nurses at the YMHH. Psychiatrists working at the research site identified caregivers based on perceived availability, willingness and ability to participate in the research, given their familiarity with the history of patients and caregivers. Caregivers were approached while they were waiting for a psychiatrist to hear about their family member’s condition or while waiting to meet the hospitalized person and invited to participate in the research. If they agreed, an appointment for the in-depth interview was scheduled. All thirty caregivers accepted the invitation to participate in this study.

### Data collection

Face-to-face in-depth interviews were conducted one-on-one with the caregivers in the Burmese language using a semi-structured data collection instrument [47]. Structured questions were used to elicit information about socio-demographic characteristics of caregivers, their families and the substance using family member, while unstructured questions focused on the socioeconomic and psychological burden of caregiving, coping methods and barriers to coping. The purpose of the study was explained to interested caregivers and written informed consent was received before the start of the interview. The interview was completed at a place convenient for the respondent and took approximately forty-five minutes to one hour. While conducting the interview, each participant was given snacks and coffee worth about 4 US dollars.

Permission to collect the data was obtained from the hospital, while ethical clearance was obtained from the Institutional Review Board, Defence Services Medical Research Centre, Directorate of Medical Services, Ministry of Defence, Republic of the Union of Myanmar on May 4, 2019 with reference number IRB/2019/15.

### Data analysis

All respondents were Myanmar nationals and the in-depth interviews were conducted, recorded and transcribed in the Burmese language. Verbatim transcriptions were prepared after each interview and subsequently translated into the English language. The data were analysed using framework analysis [48]. The transcripts were read several times to become familiar with the data and pinpoint pre-identified and new themes. Pre-identified themes were derived from the literature and comprised socioeconomic burden (financial and income losses, social limitation, family conflict) and psychological burden (sadness, anger, helplessness, fear, guilt, worry), active and avoidant coping strategies and barriers to coping (stigma, lack of support) [7, 13, 14, 28, 30, 31, 35]. Codes were developed and refined until no new codes emerged. A matrix table was then used to group codes into themes and analyse the data across cases and themes. The last step involved reviewing and refining the themes. No new themes related to caregiver burden emerged, but revisions were made to include drug use risk factors, reasons for seeking treatment at the YMHH and national drug laws and policies. National drug laws and policies were identified as an additional barrier to coping, increasing caregiver burden.

## Results

Sociodemographic characteristics of the thirty caregivers are summarized in Table 1. More than four-fifths of respondents were from Yangon and more than half were female and aged 45 years and above. Almost two thirds of caregivers were the parent of a family member with substance use disorder and had their own business. According to the respondents, the majority of substance using persons were young, male, single and unemployed. Regarding the type of illicit drugs used by the family member, amphetamine was mentioned most often, followed by heroin and cannabis. Most of the care recipients were reported to also consume other types of psychoactive substances such as tobacco, alcohol and betel, while some had co-occurring use of heroin or cannabis and amphetamine.

**Table 1:** Socio demographic characteristics of caregivers and their family members.

**Caregivers’ characteristics**	**Frequency (N)**	**Percent (%)**
**Age (years)**		
≤30	4	13.3
31–44	10	33.3
≥45	16	53.3
**Sex**		
Male	11	36.7
Female	19	63.3
**Marital status**		
Single	3	10.0
Married	24	80.0
Divorced	1	3.3
Widowed	2	6.7
**Residence**		
Yangon	25	83.3
Other regions	5	16.7
**Relationship**		
Parent	18	60.0
Son	1	3.3
Sibling	6	20.0
Wife	4	13.3
Uncle	1	3.3
**Occupation**		
Unemployed	3	10.0
Own business	19	63.3
Employee	8	26.7
**Income per month (Household)**		
≤300,000 MMK (≤200 USD)	7	23.3
300,001–700,000MMK(200–450 USD)	13	43.3
>700,000 MMK (>450 USD)	10	33.3
**Characteristics of the family member with DUD**	**Frequency (N)**	**Percent (%)**
**Age**		
≤18	3	10.0
19–24	12	40.0
25–29	5	16.7
30–34	3	10.0
≥35	7	23.3
**Sex**		
Male	29	96.7
Female	1	3.3
**Occupation**		
Unemployed	19	63.3
Own business	8	26.7
Work in family business	3	10.0
**Types of drug used**		
Amphetamine	21	70.0
Heroin	5	16.7
Cannabis	4	13.3

MMK: Myanmar kyat; USD: US dollar.

### Risk factors for drug use

Respondents felt it was important to share their opinion about the risk factors for drug use prior to discussing caregiver burden. Risk factors for drug use was a new theme that emerged from the data analysis, possibly motivated by associative stigma experienced as caregiver of a family member with DUD [49] and resulting feelings of social desirability. Easy accessibility of drugs and peer pressure were identified by several respondents as the most important risk factors. Seven caregivers pointed out that there is an abundance of illicit drugs in their community and that drugs can be obtained easily.

*“I don’t think he can withdraw from his addiction*. *In this day and age*, *drugs are very easy to get and use*, *even if there is only a little bit of money*. *I am worried about the future*. *He will use drugs again; I will have to send him to the hospital again*. *And I want to ask how long I have to survive in this circle*? *Until he dies or until I die*?*”* (28-year-old sister of a person using amphetamines)

An equal number of caregivers shared that their family member tried drugs when attending university since they met friends who used drugs. One caregiver said her child became addicted because of her partner.

“*She started this when she eloped with her boyfriend*. *The wife of an alcoholic uses alcohol*. *Like this*. *She injected when she could not protect her husband from using drugs and she became addicted herself*.” (48-year-old mother of a person using heroin)

Four caregivers said that their family member started using drugs because of family problems such as growing up in a broken family, death of the mother or not living together with their parents.

“*He became like this because of his mother’s death*. *Since I am the household head*, *I could not stay besides him*. *I had to work and I sent him to his grandmother’s house*. *So*, *there was no one to control him*. *He started to hang out with many friends and did not go back home at night*.” (64-year-old father of a person using amphetamines)

More than half of the caregivers subsequently also explained why their family member was hospitalized. Some caregivers, especially parents and siblings, said that they insisted their family member to go to the hospital since they could not control their behaviour and aggressiveness anymore. Almost one third of caregivers, however, pointed out that it was the family member’s own desire to go to the hospital and to withdraw from drugs. Underlying reasons given include an intrinsic yearning to overcome the drug problem, feeling sorry for family members, regret and fear of imprisonment. One caregiver expressed how she admired her brother’s decision to come to the hospital as follows:

“*He is an “addict”*. *Alright*. *But he tried to get rid of drugs by himself and he can still be a role model for accepting his mistake*. *We do not know how to go to the hospital*. *He drove to the hospital himself*.” (37-year-old sister of a person using amphetamines)

### Socioeconomic burden on caregivers

#### Financial costs and income losses

DUD take a heavy financial toll on the substance using person and their caregivers in terms of financial costs and income losses. The financial costs of DUD comprise drug, medical, religious, and legal costs. Depending on the type of substance use, the cost of the drug itself can be substantial. One caregiver, the mother of a person with DUD, for example, emphasized that heroin was very expensive and cost nearly 300,000 MMK (nearly 200 USD) per week. Second, almost all of the respondents incurred both direct medical and non-medical expenses when their family member was hospitalized to access treatment. Direct medical expenses other than bed charges, however, were borne by the YMHH. Hospitalization-related out-of-pocket expenses commonly encountered by caregivers, therefore, mainly included bed charges, and expenses for food and transportation. Even for caregivers living in Yangon, transportation was a major component of expenses, as the hospital is very far from the city centre. Caregivers from other regions incurred additional accommodation expenses.

“*I did not have to pay too much for hospitalization fees*, *but bed charges were 31*,*000 MMK for the two weeks*. *Since I came from Ye*, *transportation charges were so high*, *200*,*000 MMK for hiring a van and nearly 45*,*000 MMK for food*. *However*, *accommodation was just 1*,*000 MMK per day*.” (49-year-old mother of a person using amphetamines) [Ye is a township about 450 kilometres south of Yangon by road.]

“*I used to send him to a private hospital and it cost 1*,*500*,*000 MMK just for one month of hospitalization*. *Millions of money were lost for his drug problem…*” (40-year-old wife of a person using amphetamines)

In addition, a few respondents said that they also paid for religious activities, encountered legal fees and/or had to settle third-party compensation claims related to DUD.

*“It has been nearly two years since I attended a court hearing and the case has not been settled yet*. *Over 1*,*000*,*000 MMK were spent on his legal case*. *And I had to pay money to the police whenever they arrested my son for sitting in the dark*, *at night*, *in front of other peoples’ homes*. *I am very worried that I could not afford the expenses for him any more if I do not have money*.*”* (46-year-old mother of a person using amphetamines)

Lost income due to caregiving mainly comprised time missed from work and activity restrictions. The income loss associated with accompanying their family member to the hospital was higher for the 63 percent of caregivers, who had their own business, compared to employed caregivers who received relatively low wages.

*“I had to close my business*, *took him to the hospital and stayed all day and night besides him*. *It is more tiring than working*. *Actually*, *I can get approximately 40*,*000 MMK per day if I work*.*”* (40-year-old father of a person using cannabis)

Not only caregivers, but also persons with DUD experienced income losses. A few caregivers pointed out that their family member is not interested in working any more, but one caregiver added that persons with DUD, even if willing to work, face job limitations.

*“He tried to find work but he couldn’t because of his appearance*. *He is so thin and unclean*, *like a “drug addicted street boy”*, *and he also has tattoos on his body*. *You see*! *Which employer wants to give him a position*? *Before starting to use drugs*, *he worked*. *Nearly 100*,*000 MMK of his income were lost and this made it more difficult for me to survive*.*”* (52-year-old mother of a person using amphetamines)

Selling the family’s property and pawning were linked with income losses since caregivers had to sell income generating assets to solve the financial problems related to their family member’s drug use.

“*I owned a house in the past and I opened a grocery store*. *My income was 300*,*000 MMK per month*. *Now*, *it has been sold and I get only 100*,*000 per month in the new place since there are only a few customers*. *I also pawned my jewellery*.” (46-year-old mother of a person using amphetamines)

A strong relationship between incurring financial losses and caregivers’ emotional feelings emerged. Seven caregivers broke into tears during the interview saying that their financial crisis made them feel so sad, depressed and worried about the future.

“*She worked and looked after the family before becoming addicted*. *Since then*, *the house has been sold to be able to pay off some of the debt*. *I am drowning in problems since I have to spend extra money*, *also for her partner who is an addict as well …*” (48-year-old mother of a person using heroin)

#### Social limitation

While caregivers did not explicitly state that they developed social withdrawal because of their family member’s DUD, five caregivers, especially parents, mentioned that they could not participate in any social activities any more since most of their time was devoted to their family member’s well-being and related activities such as cooking, accompanying the family member to the hospital and watching over the family member to prevent drug use.

“*I cannot go to the monastery*, *even during the Thingyan Holidays as I do care and cook for him*. *For a long time*, *I have not been able to attend weddings and other social activities*.” (49-year-old mother of a person using amphetamines) [The Thingyan Holidays are the New Year Holidays in Myanmar.]

#### Negative impact on family relationships

Almost one third of the respondents pointed out that the DUD of the family member negatively affected other family members and family cohesion. Stealing to be able to purchase drugs was a common behavioural problem faced by caregivers and other family members. The family member with DUD threatened other family members when asking for money and sometimes they pawned things, which increased the indirect financial cost of the household and destroyed family relationships. One female caregiver described that her brother even tried to take the money she put aside for religious offerings.

*“If there are only three persons at home*, *the money did not disappear—no matter where I placed it*. *But if he is at home*, *even the money I had hidden disappeared*. *He stole the money which I intended to donate for monks and god*. *See*! *How stupid he is*.*”* (28-year-old sister of a person using amphetamines)

Two caregivers, both mothers of persons with DUD, said that they neglected other family members because of taking care of the substance using family member. One caregiver explained that her husband’s drug use problems affected their child.

“*He jeopardizes our child’s future*, *education and also our business*. *He even tried to take money from his son’s tuition fees*, *even though I told him that this is for education*. *I am worried that my adolescent (son) imitates his father*. *My son is in grade 11 and he always goes to school and tuitions*. *He returns late at night and it is a dangerous condition for him as he risks being wrongly accused under drug laws by the police*.” (40-year-old wife of a person using amphetamines)

### Psychological burden on caregivers

Emotional distress was widespread among caregivers. Almost all respondents said they experienced emotional distress when taking care and dealing with the behavioural problems of their family member with DUD. Common negative emotions included sadness, anger, helplessness, fear, worry and guilt. More than half of the caregivers, especially mothers, felt sad and hurt of seeing their loved one suffering from DUD.

*“I have no tears left to cry*. *If I could*, *I would like to exchange her blood for new cells*. *She used to be afraid of syringes and she never had injections*, *even when she was ill*. *I cannot imagine how she injected heroin by herself*. *She is always asking for drugs*. … *What can I do*… *I …*.*”* (48-year-old mother of a person using heroin)

Parents expressed feelings of guilt and hopelessness for their children and some reported feelings of shame together with anger when their children became rude and physically abusive towards them.

*“I have never been optimistic about my son*, *because he uses drugs*. *I have always scolded him and he may not have wanted to live at home*. *I regretted that when seeing the psychiatrist speaking to him kindly and guiding him patiently*. *I have never spoken to my son like this*. *I am guilty and I could not provide enough support for his education because of my low salary…”* (52-year-old mother of a person using amphetamines)

“*I was very angry and also ashamed when he did not recognize me as his mother because of the strong effect of the drug*. *He pulled my hair hard and I thought that it is better for him to die or for me to escape from this retribution*.” (46-year-old mother of a person using amphetamines)

The feeling of helplessness was emphasized by three caregivers who were the main breadwinners of the family.

“*I cannot speak out loud about these difficulties*, *neither to my mom nor others*, *because it is an abnormal situation*. *I wish I was mad*. *I have to run the business by myself*, *take care of children and take care of him as well*. *How can I handle all this by myself*?” (40-year-old wife of a person using amphetamines)

Some caregivers were worried that their family member would use drugs again upon discharge from the hospital. Moreover, nearly two thirds of respondents, especially female caregivers, reported feelings of fear. The largest fears concerned injuries inflicted by substance using persons on family members and others, legal problems and negative judgements from people in their surroundings.

*“I have heard in the news that a “drug addicted person” killed a family member or a person in the neighbourhood after an overdose of drug and I am afraid of him causing danger to himself or to me or my family*.*”* (28-year-old sister of a person using amphetamines)

Caregivers also pointed out that magnitude and characteristics of the psychological burden were affected by the nature of the relationship between caregiver and family member, as well as between other family members and the family member with DUD. While negative relationships were perceived by respondents to exacerbate the psychological burden, positive attitudes were thought to lower it. Two caregivers added that caregiving also had negative effects on their physical health. They reported suffering from sleep disturbances, since they were too worried about their family member, and increased physical health problems such as hypertension, diabetes and heart diseases which were associated with stress.

### Coping strategies

Coping strategies can be classified as active coping and avoidant coping [50]. Most respondents used active coping, whilst only a few revealed that they tried to disengage from the problem. Engaging in religious activities, accepting the situation, seeking information, planning for the future and financial coping are active coping strategies used by caregivers to tackle the challenges associated with caring for persons with DUD. As most caregivers interviewed were Buddhists, religious activities included prayers, paying homage to Buddha, becoming vegetarian and performing religious activities for their family member, as well as for the peace of their mind. Three caregivers made offerings to spirits, while two sought advice from fortune tellers. One of the two Muslim caregivers described that she sent her husband to Saudi Arabia to perform the Hajj pilgrimage.

“*With the intention of freeing him from drugs*, *I sent him to do the Hajj and to perform religious rites in Saudi Arabia*. *However*, *he came back from the Hajj*, *used the drug and the cycle started again*. *I remembered that nearly 6*,*000*,*000 MMK were paid for this activity*.” (40-year-old wife of a person using amphetamines)

“*If someone else was in my shoes*, *they would have become mad*. *I have to take care of the whole family*, *including him*. *Sometimes I became angry with him and sometimes I wanted to die*. *But gradually*, *I tried to devote my mind to religious activities*. *Now I am more peaceful than before and I can think that it is just fate that I cannot change*.” (47-year-old sister of a person using cannabis)

Half of the caregivers said that they accepted their family member’s problematic behaviours such as stealing and breaking things and believed their situation to be a karmic retribution. Several caregivers tried to understand their family member better, and some mentioned that they tried to obtain relevant information from television, radio and social media.

*“Previously*, *I did not know what this drug addiction means*. *But*, *I watched educational programs for DUD on TV and also read online news*. *It allows me to improve my knowledge about the disease*. *You cannot be too serious and angry with drug users*. *There is a technique to “control” them*.*”* (47-year-old sister of a person using cannabis)

Caregivers also made plans for the future of the person with DUD such as sending their family member to a rehabilitation unit after leaving the hospital, encouraging them to work or continue studying and spending more time with them.

Most of the respondents also used financial coping strategies such as selling properties, pawning, borrowing money and cutting other expenditure to cope with financial hardship.

“*I hardly ever buy clothes for myself*. *I tried to be in a good relationship with doctors and nurses at the hospital*. *I work and I wear what they give to me*.*”* (52-year-old mother of a person using amphetamines)

Avoidant coping strategies such as denial, behavioural disengagement (i.e. reduced efforts to deal with caregiving [51]) and alcohol or drug use were hardly used by respondents. Only three caregivers said that they thought about disengaging from the stressful situation and the large responsibility. Yet, no one had ever abandoned their substance using family member. Also, substance use (alcohol and sleeping pills) was rarely used as a coping method to deal with problems. Two male respondents said that they drank some alcohol when they were tired and annoyed though.

### Barriers to coping

The two main barriers to active coping respondents identified were stigma and the lack of support [31, 35, 36].

#### Stigma

Nearly one third of the respondents said that they were exposed to stigma associated with DUD and related negative sentiments from relatives, neighbours and friends. Stigma is perceived to be a barrier to financial coping and seeking social support. Especially mothers were blamed for their child’s DUD.

*“People blamed me that letting my son become an “addict” was my fault and that I destroyed him by giving money*. *There is no mother who gives their children money for drugs*. *There is no mother who encourages them to use drugs*.*”* (40- year-old mother of a person using amphetamines)

Furthermore, caregivers were looked down upon by people in their surroundings because of their family member’s substance use and impolite behaviours. Family members were labelled as father, mother or sibling of the “junkie”. Three caregivers said that even very close relatives tried to end their relationship since they are worried about their own children imitating the behaviour of the person with DUD, increasing social isolation.

*“I am left behind by the society*. *There was a person who graduated with a specialization in law and he said that if someone is a criminal*, *the police will arrest only this person*. *However*, *if the suspect is involved in drug cases*, *the whole family cannot escape*.*”* (52-year-old mother of a person using amphetamines)

Associative stigma exacerbated the social isolation of caregivers and was a barrier to seeking emotional support. Moreover, some caregivers emphasized that the persons with DUD themselves were also exposed to high levels of stigma.

*“What can I say*? *He has no future*. *He has been judged as “junkie” by his colleagues*. *I am afraid that he cannot enter into society again*. *I am very depressed of seeing these things…*.*”* (29-year-old wife of a person using amphetamines)

#### Lack of support

Almost all of the caregivers said that they did not receive any help from outside the family to deal with the wide range of issues. One caregiver shared that even the local authority failed to issue the referral documents in time to send the substance using family member to the hospital and gave wrong advice.

*“Family members help each other but there is a lack of support even from the local authority*. *I was greatly surprised by his suggestion*, *because he told us to give alcohol or sleeping pills for my patient to control aggressiveness instead of sending him to the hospital*.*”* (28-year-old sister of a person using amphetamines)

One caregiver from Kayin State (about 300 kilometres away from Yangon by road) pointed to supply-side barriers and said that she had to travel to Yangon because of the shortage of treatment centres in Kayin State. A few caregivers stressed financial problems and suggested that it would be good to have charitable organizations at the existing mental health hospitals to provide financial support for medication and other necessities for persons with DUD.

Another caregiver drew attention to the legal framework by expressing his disappointment with the existing drug law and policy as follows:

*“The government should place more emphasis on developing supporting programmes instead of making the policy to be harsh*. *The police imprison offenders with only two or three amphetamine-type stimulants in hand while large scale traffickers still escape*. *It should not be*…*”* (64-year-old father of a person using amphetamines)

Surprisingly, half of the caregivers who discussed the lack of support indicated that they did not hope for any support from anywhere because they thought that it was not the responsibility of the public to help and that it was shameful to talk about this openly. Some even stated that it was not worthwhile for the government to support persons with DUD.

“*I don’t think the government will support drug users*. *It’s funny*, *isn’t it*? *So*, *when your child becomes addicted*, *it is totally your responsibility*. *It is too shameful to admit even to close relatives*. *So*, *how could I announce this to the public*? *I don’t want any support for this case*.” (57-year-old father of a person using amphetamines)

## Discussion

DUD impose a burden on substance using persons, their family members, and family caregivers [7, 31, 35, 38]. Yet, evidence is limited, especially for Southeast Asian countries. This study focused on Myanmar, a country where drug use has been criminalized, and analysed qualitative data from thirty informal caregivers of persons with DUD, who were treated at the YMHH, one of Myanmar’s two tertiary-level mental health hospitals, to better understand the burden they are facing, coping strategies used and barriers to coping.

Caregivers highlighted the importance of background as well as context and argued that it is important to understand the factors underlying substance use. These included peer pressure, family problems and easy access to drugs in the neighbourhoods despite the country’s harsh drug law. Peer pressure was also identified as a key risk factor in Sibeko, et al. [52] and a source of worry as caregivers were concerned about the continued influence of peers on their family member’s behaviour. Most caregivers further explained that they went to the YMHH, because of their family member’s desire to withdraw from drugs which is in line with recovery-oriented approaches. Evidence, however, suggests that persons with DUD often do not seek speciality care on their own [53]. These new themes, drug use risk factors and reason for seeking treatment, while not in line with the study’s primary objective, may have been driven by caregivers’ desire to garner some social approval, given the high level of stigma persons with DUD and their families experienced.

Since 1993, when cost-sharing was introduced, out-of-pocket health expenditures have been the main source of finance in Myanmar, accounting for 79 percent of total health expenditure in 2011 [54]. Only 0.3 percent of total health expenditure are used for mental health [55, 56] and out-of-pocket expenditures on treatment of DUD are high. The mental health treatment gap is estimated to be almost 90 percent due to factors such as stigma and insufficient knowledge about mental disorders, despite the fact that community mental health care has been developed since 1990 [54]. Therefore, the bulk of the financial and non-financial burden related to DUD is typically borne by the substance using person and their family members, which is echoed by the findings in this study. All respondents were financially affected as they had to pay for direct medical and non-medical expenses such as bed charges, and expenses for food and transportation. Transportation cost were not only high for caregivers living outside of Yangon, but also for caregivers residing in the country’s largest city since the hospital is located far away from the city centre. Iseselo, Kajula, & Yahya-Malima [30] also found that most caregivers lived far away from the hospital and faced transportation challenges, which prevented some from going to the hospital. Moreover, caregivers living outside of Yangon had to bear additional accommodation expenses. Other expenses included legal fees, payments to police officers and expenses for religious rituals. In addition, caregiving was found to result in substantial indirect cost. Prior research also found that caregivers faced income losses as they either had to cut back working hours or were unable to work due to the responsibilities associated with caregiving [20, 30].

Moreover, taking care of persons with mental disorders limits social interactions and can result in social isolation and withdrawal [28, 30]. Consistent with the literature, some caregivers felt socially isolated due to their caregiving responsibilities, which prevented them from participating in social activities. Moreover, caregiving and harmful behaviour of persons with DUD such as stealing and physically or verbally abusing family members were found to negatively affect family relationships. Usher, et al. [31] also found that stealing from family members was a common problem of substance use.

As expected, caring for a person with DUD was found to negatively affect respondents emotionally. Most caregivers expressed sadness about seeing a loved one ill and their hope for the family member’s future shattered. This linkage between caregiver’s grief and a family member’s mental health problems was found in several studies [26–28, 31, 35]. Feelings of grief, hopelessness and guilt were particularly pronounced among parents, who were taking care of a child with DUD. Moreover, feelings of guilt were stronger in case of poor caregivers as they thought that they could neither support their family member well enough financially nor devote sufficient time to provide care, given that they have to work to survive. Feelings of guilt related to caregiving were also discussed in prior work [25–27, 31]. Behavioural problems of persons with DUD triggered feelings of sadness and anger in caregivers, which supports that behavioural problems of patients are predictors of negative emotions in caregivers [7, 32].

To deal with these challenges, most of the respondents adopted active coping strategies such as performing religious activities, accepting their situation and planning ahead, which is consistent with studies that analysed the coping mechanisms of caregivers of persons with schizophrenia and breast cancer [50, 57]. Furthermore, some of the caregivers would like their family member to use religious coping strategies after being discharged from the hospital and to spend time in a monastery. This is similar to the findings of a study in Ghana, where caregivers sent their mentally ill relatives to prayer camps [28]. Financial coping strategies used by caregivers who participated in this study comprised selling belongings, pawning assets, borrowing money from relatives and others, as well as cutting other expenditures, which is likely to worsen their situation. Reducing other expenses is a common coping strategy to deal with financial difficulties [28, 30]. Given that no public health education programmes about DUD were offered at the community level and the perceived need for information, caregivers tried to obtain related information from other sources such as television, radio and social media, which helped them to better understand DUD and how to deal with these. Similar mechanisms are reported in Shamsaei, Cheraghi, & Esmaeilli [26].

Family caregivers’ ability to cope with their situation, however, was found to be impeded by stigma and a lack of support. Caregivers experienced associative stigma and explained that not only persons with DUD but also caregivers were discriminated against by their surroundings and close relatives. This is consistent with findings from Australia, where parents of substance using persons had to endure blame and criticisms from their neighbours [31]. The 2018 National Drug Control Policy explicitly recognizes that “*Drug dependency is rarely appreciated as a health issue*, *and drug users in Myanmar face stigma*, *social exclusion and limited access to services*.” [41, p. 8] According to the Burnet Institute, needle and syringe exchange programmes for injecting drug users, based on harm reduction approaches, had to be excluded from the services offered by their drop-in centres in Yangon, because people were afraid of having these centres in their surroundings [58]. A few caregivers indirectly pointed to the existing drug laws and explained that these increase stigma as people are afraid of the police, the court of law and imprisonment. According to the 1993 Narcotic Drugs and Psychotropic Substances Law, a person is sentenced to about five to ten years in prison if he or she is found to possess only a small amount of illicit drugs [39]. Almost 50 percent of all prisoners countrywide serve sentences for drug-related offences [41] in prisons that are known for their appalling conditions [59]. Yet, most large-scale drug traffickers have remained unscathed [39], which a few respondents also pointed out. The country’s drug laws and policies have traditionally focused on repression and punishment, undermining the human rights of those affected [60]. In 2018, the National Drug Control Policy was announced, heralding a shift from a punitive and coercive towards a harm reduction approach to drug use [43]. Yet, the 2018 National Drug Control Policy needs to be supported by further amendments of the 1993 Narcotic Drugs and Psychotropic Substances Law to ensure that standards of proportionality in sentencing for drug offences are met as discussed in Cachia [43]. In Myanmar, support from the government and other organizations for people with DUD, their caregivers, as well as other family members is inadequate at all levels. Supply side factors increased the socioeconomic and psychological burden of households with persons experiencing drug dependence. There are only two tertiary-level mental health hospitals and most of the drug treatment centres in other states and regions are not functioning well as evidenced by their low utilization rates [61]. Similar findings were reported in rural Ghana, where caregivers did not receive financial and social support at the community level because of poverty, lack of sympathy and high stigmatization [28].

## Suggestions for easing caregivers’ burden

The findings in this study show that caring for persons with DUD imposes a tremendous burden on informal caregivers and their families, which is amplified by the country’s harsh drug laws, drug-related stigma and the lack of support. Given that less than one percent of government health expenditures is used for mental health, mental health expenditures need to be increased to improve treatment and rehabilitation services and ease caregivers’ financial burden. The results of this study further underline that a shift from a punitive approach to a health-focused and evidence-based harm reduction approach to deal with drug-related challenges is urgently needed as envisaged in the 2018 National Drug Control Policy. To facilitate harm reduction approaches, interventions such as targeted education campaigns to reduce the stigma associated with drug use and DUD should be prioritized. Decriminalizing drug users would help promote human rights, reduce stigma and social exclusion, and thereby ease caregivers’ burden. Moreover, reducing the stigma enshrined in the current drug law is likely to facilitate access to treatment and rehabilitation services. To increase society’s understanding of DUD-related issues, including treatment and rehabilitation services, public health education efforts could be strengthened. The study by Orford, Templeton, Patel, Copello, & Velleman [34] showed that health education can also improve the coping skills of caregivers.

## Limitations

It is important to point out that this study has several limitations. First, only caregivers of persons who received treatment at the YMHH were included, which introduced a bias. The YMHH is one of the country’s two tertiary-level mental hospitals and, therefore, a flagship hospital. In addition, the burden caregivers are facing is likely to be largely underestimated as caregivers of those persons with DUD who do not receive treatment or caregivers of imprisoned persons with DUD were not included. The small sample size did not permit a clear distinction between caregivers of persons with DUD by type of substance use (amphetamine, heroin and cannabis). Also, as receiving treatment entailed registration of the family member as a “drug user” at the Ministry of Health until 2018 [43], respondents may have been afraid of voicing all their concerns due to fears of prosecution despite the registration amnesty. Last not least, given that this study uses a qualitative research design, the findings cannot be generalized.

## Future research

Future studies should consider a wide range of caregivers, including caregivers of patients who receive treatment at facilities other than the YMHH, patients who do not receive any treatment, and patients who are imprisoned. These studies should adopt a quantitative or mixed methods research approach to better understand the situation of caregivers and complement the findings of this research. A larger sample size would also permit an analysis by type of substance use. Last not least, while none of the caregivers who participated in this study pointed to positive experiences from caregiving, possible benefits of caring for patients with DUD could be explored.

## Conclusion

Myanmar has been facing extensive drug-related challenges, including widespread drug use despite the fact that substance users continue to face five to ten years of imprisonment. The results of this study suggest that the socio-economic and psychological burden of caring for patients with DUD is tremendous in Myanmar. Socio-economic burden consists of financial constraint, income loss, social limitation and negative impact on family cohesion. Sadness, anger, helplessness, worry, fear and guilt, on the other hand, are the main psychological distress factors. Most caregivers used active coping rather than avoidant coping, but barriers to coping exist. These include perceived stigma towards persons with DUD and their caregivers and a lack of support. Both are amplified by the country’s current drug law, highlighting that the 1993 Narcotic Drugs and Psychotropic Substances Law should be revised further.
